# Trends of sputum-smear positive tuberculosis in Zimbabwe: 2008–2011

**DOI:** 10.1186/s13104-015-1568-z

**Published:** 2015-10-16

**Authors:** Grace Noppert, Zhenhua Yang, Charles Sandy, Joconiah Chirenda

**Affiliations:** Department of Epidemiology, School of Public Health, University of Michigan, Ann Arbor, MI USA; Department of Community Medicine, College of Health Sciences, University of Zimbabwe, 3rd Floor New Health Sciences Building, Parirenyatwa Hospital Complex, Avondale, P O Box A178, Harare, Zimbabwe; National TB Programme, AIDS and TB Unit, Ministry of Health and Child Care, Harare, Zimbabwe

**Keywords:** Trends in tuberculosis, Sputum smear positive tuberculosis, Zimbabwe

## Abstract

**Background:**

Tuberculosis (TB) has remained one of the major public health problems in Zimbabwe with an estimated incidence rate of 552 per 100,000 persons in 2013. The aim of this study was to describe the trends in acid-fast bacilli (AFB) sputum-smear positive (SSP) TB overall and within subpopulations for the period during 2008–2011 in Zimbabwe. Results of this study will contribute towards the evaluation and implementation of targeted TB control interventions.

**Methods:**

A cross-sectional study design was used to analyze 40, 110 SSP TB patient records routinely collected during 2008–2011. Incidence trends of SSP TB were described by province, sex, and age group. A Mantel–Haenszel Chi Statistic was calculated to compare each provincial SSP TB notification rate to the national SSP TB notification rate.

**Results:**

SSP TB notification rates were higher in the two main urban provinces, the western provinces and Manicaland. The 25–44 year age group accounted for the largest proportion of notified SSP TB. However, the 55–64 year and 65+ age groups had SSP TB notification rates in 2011 higher than the 2008 value. Finally, the average SSP TB notification rate in males was 23 % higher than in females.

**Conclusion:**

The findings of this study suggest that TB control has successfully decreased the notification rate of SSP TB in Zimbabwe during 2008–2011. However, the disproportionate distribution of SSP TB among different regions and subpopulations of the country highlights the need for more targeted interventions to accelerate the decline of TB in Zimbabwe.

## Background

Despite tuberculosis (TB) being a long-standing, worldwide disease the global burden of disease attributable to TB continues to be a major public health concern. In 2013 alone there were an estimated 9.0 million new cases of TB worldwide and 1.5 million deaths attributable to TB, 80 % of which were occurring from 22 high-burden countries (HBCs) [1].

With a population of 12.9 million people and a national estimated TB incidence rate of 552 per 100,000 persons in 2013, Zimbabwe is among the 22 HBCs [2, 3]. In 2010–2011, Zimbabwe reported a generalized human immunodeficiency virus (HIV) prevalence of 15 % among the entire population [4]. Among TB patients with known HIV status, 70 % were HIV-positive in 2012 [1]. These data clearly show TB is a critical public health issue in Zimbabwe.

Over the last two decades, Zimbabwe has also experienced severe socio-economic challenges resulting in a substantial migration of citizens to neighboring countries, mainly the Republic of South Africa, Botswana, and Namibia. These countries have reported high prevalence rates of TB and drug-resistant TB [5]. Describing the epidemiology of TB in the Zimbabwean context, especially after the events of recent years, is essential to the development of more targeted interventions in high TB transmission zones and within vulnerable subpopulations.

The World Health Organization (WHO) reports the overall burden of TB as well as that of sputum-smear positive (SSP) TB, representing the cases at high risk for transmission within a population. It is imperative to know the trends in SSP TB within subpopulations of a country in order to develop more targeted TB control strategies for the country. To address these knowledge gaps in regards to SSP TB we used national TB notification data to analyze trends in the acid-fast bacilli (AFB) SSP TB notification rate by province, sex, and age from 2008 to 2011 in Zimbabwe.

## Methods

### Study population and data sources

We used aggregated TB notification data collected by the Zimbabwe Ministry of Health’s National TB Programme (NTP) during 2008–2011. The study sample included all new and retreatment (those who were previously cured) AFB SSP TB cases diagnosed during the study period as measured by the notification data [5–7]. Permission to use the routinely collected data was obtained from the Ministry of Health’s NTP. We focused our study only on SSP TB cases as they represent the most infectious TB cases and are therefore a priority area for TB control [8]. Country level background information was ascertained from the Zimbabwe Demographic Health Survey from 2010 to 2011 [4]. The population size data was extracted from the 2002 Population Census carried out by Central Statistical Office of Zimbabwe [2, 9].

There were a total of 164,535 cases of TB reported from 2008 to 2011 in Zimbabwe. Of these 164,535 reported cases, 141,104 (86 %) were pulmonary TB cases and 23,432 (14 %) were extra-pulmonary TB (EPTB) cases. Of the 141,104 pulmonary cases, 130,000 were new cases and 11,104 were retreatment cases. Sputum-smear results were available for 104, 123 (74 %) of the pulmonary cases. Among the new pulmonary cases, 30 % were SSP, 36 % sputum-smear negative (SSN), and 34 % sputum-smear not done. Among the retreatment pulmonary cases, 10 % were SSP, 28 % SSN, and 62 % sputum-smear not done. The high proportion of sputum-smear not done among the retreatment cases was due to loss of experienced health care workers to neighboring countries, inadequate diagnostic capacity and reduced funding. Our final analysis included 39,000 new SSP pulmonary cases and 1, 110 retreatment SSP pulmonary cases for a final sample size of 40, 110 cases.

### Statistical analysis

We described the trends of SSP TB by province, sex, and age group. For the purposes of our analyses Chitungwiza and Harare Cities were analyzed as one province. Chitungwiza City is 20 km from Harare and has been the dormitory city for Harare’s workforce. Most TB cases from Chitungwiza City were diagnosed, treated, and reported in Harare during the study period.

A Mantel–Haenszel Chi Statistic was calculated to compare each provincial SSP TB notification rate to the national SSP TB notification rate. All statistical analyses were carried out in Microsoft Excel (2011). We used the average SSP TB notification rate over the four-year period to classify provinces as having an SSP TB notification rate either above or below the national average SSP TB notification rate. We then created a map comparing the average SSP TB notification rate for each province to the national average SSP TB notification rate.

## Results

### National trends of SSP TB cases

In 2008, 10,511 cases of SSP TB were reported by the Zimbabwe NTP. There was a spike in the number of SSP TB cases reported in 2010 to 12,991 cases. By 2011, the number of SSP TB cases had dropped to 10,082: a reduction of 22.39 % from 2010. However, this was only a 4.08 % reduction comparing the 2011 SSP TB case number to the 2008 SSP TB case number. These data correspond to an increase in the national SSP TB notification rate from 90.37 per 100,000 persons in 2008 to 111.69 per 100,000 persons in 2010 followed by a decline to 86.68 per 100,000 persons in 2011 (Table 1).

**Table 1:** The notification rate of sputum-smear positive tuberculosis per 100,000 persons in Zimbabwe during 2008–2011 for each of the 10 provinces and the nation as a whole

Province	2008	2009	2010	2011
Harare/Chitungwiza City	139.02**	133.80**	140.97**	106.85**
Bulawayo	131.53**	119.71**	165.37**	117.20**
Matabeleland North	105.54**	93.91	124.69*	95.33*
Matabeleland South	146.24**	113.47**	146.24*	124.03**
Masvingo	57.18**	59.53**	78.76**	68.92**
Mashonaland West	50.22**	48.09**	93.09**	93.66*
Mashonaland East	60.32**	96.86	102.27*	76.64**
Mashonaland Central	80.37*	91.82	118.64*	82.38
Midlands	76.91**	84.77**	92.69**	61.75**
Manicaland	116.51**	119.00**	95.22**	72.41**
National average	90.37	96.62	111.69	86.68

* A *p* value less than 0.05 based on a Mantel–Haenszel Chi Square Test comparing the provincial notification rate to the national notification rate

** A p-value less than 0.01 based on a Mantel–Haenszel Chi Square Test comparing the provincial notification rate to the national notification rate

### Trends of sputum-smear positive SSP TB by age group

The distribution of SSP TB cases in each age group remained relatively stable over the study time period (Table 2). Around 60 % of the total SSP TB cases were in the 25–44 age group (N = 28, 788), with a SSP TB notification rate of 260.2 per 100,000 persons in 2008 and declining to 244.0 per 100,000 persons in 2011. The 15–24 and 45–54 age groups accounted for the next largest proportions of total SSP TB cases at around 10 % each of the total SSP TB cases (N = 6354 for 15–24 age group; N = 5069 for 45–54 age group).The younger age groups (0–4, 5–14, 15–24) exhibited the lowest SSP TB notification rates across the entire study time period.

**Table 2:** The case number and percentage of notified sputum-smear tuberculosis cases by province, sex, and age group in Zimbabwe during 2008–2011

	2008	2009	2010	2011
Case number (%)				
Province				
Harare/Chitungwiza City	2636 (24)	2537 (23)	2673 (21)	2026 (20)
Bulawayo	890 (8)	810 (7)	1119 (9)	793 (8)
Matabeleland North	744 (7)	662 (6)	879 (7)	672 (7)
Matabeleland South	955 (9)	741 (7)	955 (7)	810 (8)
Masvingo	755 (7)	786 (7)	1040 (8)	910 (9)
Mashonaland West	615 (6)	589 (5)	1140 (9)	1147 (11)
Mashonaland East	680 (6)	1092 (10)	1153 (9)	864 (9)
Mashonaland Central	800 (7)	914 (8)	1181 (9)	820 (8)
Midlands	1126 (10)	1241 (11)	1357 (10)	904 (9)
Manicaland	1828 (17)	1867 (17)	1494 (12)	1136 (11)
Sex				
Males	5906 (54)	5933 (53)	6908 (53)	5556 (55)
Females	5126 (46)	5306 (47)	6083 (47)	4526 (45)
Age group				
0–4	47 (0.4)	51 (0.5)	41 (0.3)	41 (0.4)
5–14	283 (2.6)	277 (2.5)	305 (2.3)	219 (2.2)
15–24	1631 (14.8)	1527 (13.6)	1771 (13.6)	1425 (14.1)
25–44	6913 (62.7)	7173 (63.8)	8219 (63.3)	6483 (64.3)
45–54	1329 (12.0)	1273 (11.3)	1459 (11.2)	1008 (10.0)
55–64	506 (4.6)	579 (5.2)	712 (5.5)	538 (5.3)
65+	323 (2.9)	359 (3.2)	484 (3.7)	368 (3.7)

**Fig. 1:**
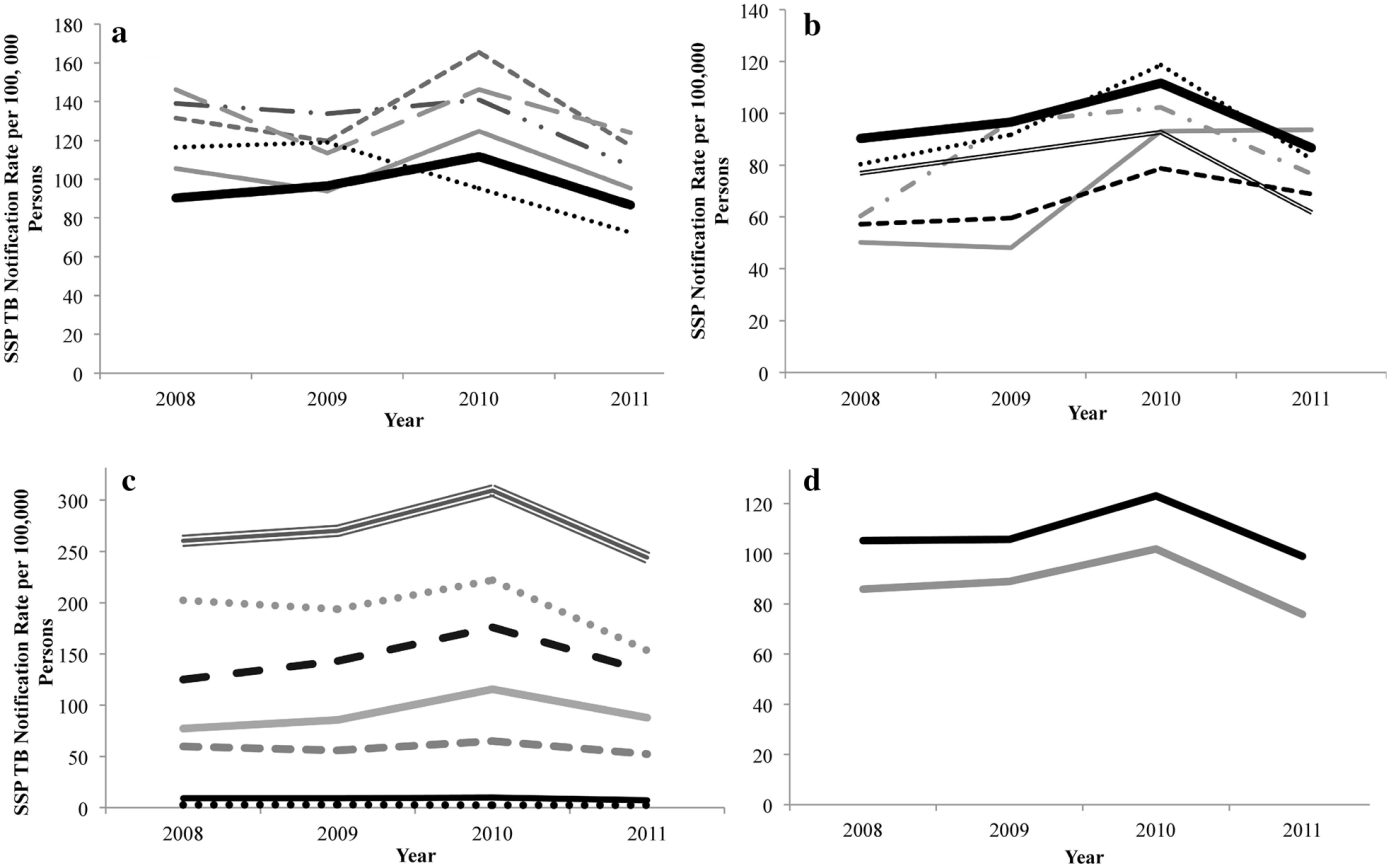
The notification rate of sputum-smear positive (SSP) TB in Zimbabwe during 2008–2011 by provinces with SSP TB notification rates above the national average (**a**), provinces with SSP TB notification rates below the national average (**b**), age (**c**), and sex (**d**) during 2008–2011. **a** Harare/Chitungwiza City; , Bulawayo; ; Matabeleland South; , Manicaland; , Matabeleland North; , National. **b**  Mashonaland West; Mashonaland East; , Mashonaland Central; , Midlands; , Masvingo; ,National. **c** , 0–4; , 5–14; , 15–24; , 25–44; , 45–54; , 55–64; ,65+. **d** , Males; , Females

There was an overall decline in the SSP TB notification rate over the study period with the exception of the older age groups of 55–64 and 65+ (Fig. 1c). The older age groups were particularly noteworthy as they had a SSP TB notification rate higher in 2011 than the 2008 rate. The 55–64 age group had an SSP TB notification rate of 125.0 in 2008 compared to 132.0 per 100,000 in 2011; the 65+ age group had an SSP TB notification rate of 77.14 in 2008 compared to 87.88 per 100,000 persons in 2011. The general decline in SSP TB case notification observed in all age groups after the 2011 spike was not significant in the older age groups.

### Trends of SSP TB by sex

There were 24, 303 (54 %) male SSP TB cases and 21,041 (46 %) female SSP TB cases. The distribution of SSP TB cases by sex was stable over the study time period (Table 2). The average SSP TB notification rate in males was 23 % higher than that of females. Males had an average SSP TB notification rate of 108.21 per 100,000 persons while females had an average of 88.12 per 100,000 persons (Fig. 1d). Additionally, females had a higher percent decline comparing the 2011 SSP TB notification rate to the 2008 notification rate; the SSP TB notification rate in females declined by 11.7 % while males only declined by 5.9 %.

### Trends of SSP TB by province

Zimbabwe is divided into ten administrative provinces, which include two urban provinces and eight rural provinces. Provincial SSP TB notification trends by year showed variation both between provinces and with the national notification trends (Table 1). In 2009, the SSP TB notification rates in Matabeleland North, Mashonaland East and Mashonaland Central were similar to the national notification rate. However, in 2011 only Mashonaland Central had a SSP TB notification rate similar to the national level. Harare province and Chitungwiza City reported higher SSP TB case notification rates than the national average since 2008 despite the actual caseload decreasing in these cities over the period (2008–2011) (Table 2).

Five provinces had an average SSP TB notification rate above the national average; the other five provinces had an average SSP TB notification rate below the national average (Fig. 1a, b). Of the five provinces that had an average SSP TB notification rate above the national average, four showed similar epidemic trajectories to the national trend. The remaining province, Manicaland, showed a different trend than the national trend. The SSP TB notification rate of Manicaland started above the national average but spiked in 2009 and decreased to below the national average by 2011.

**Fig. 2:**
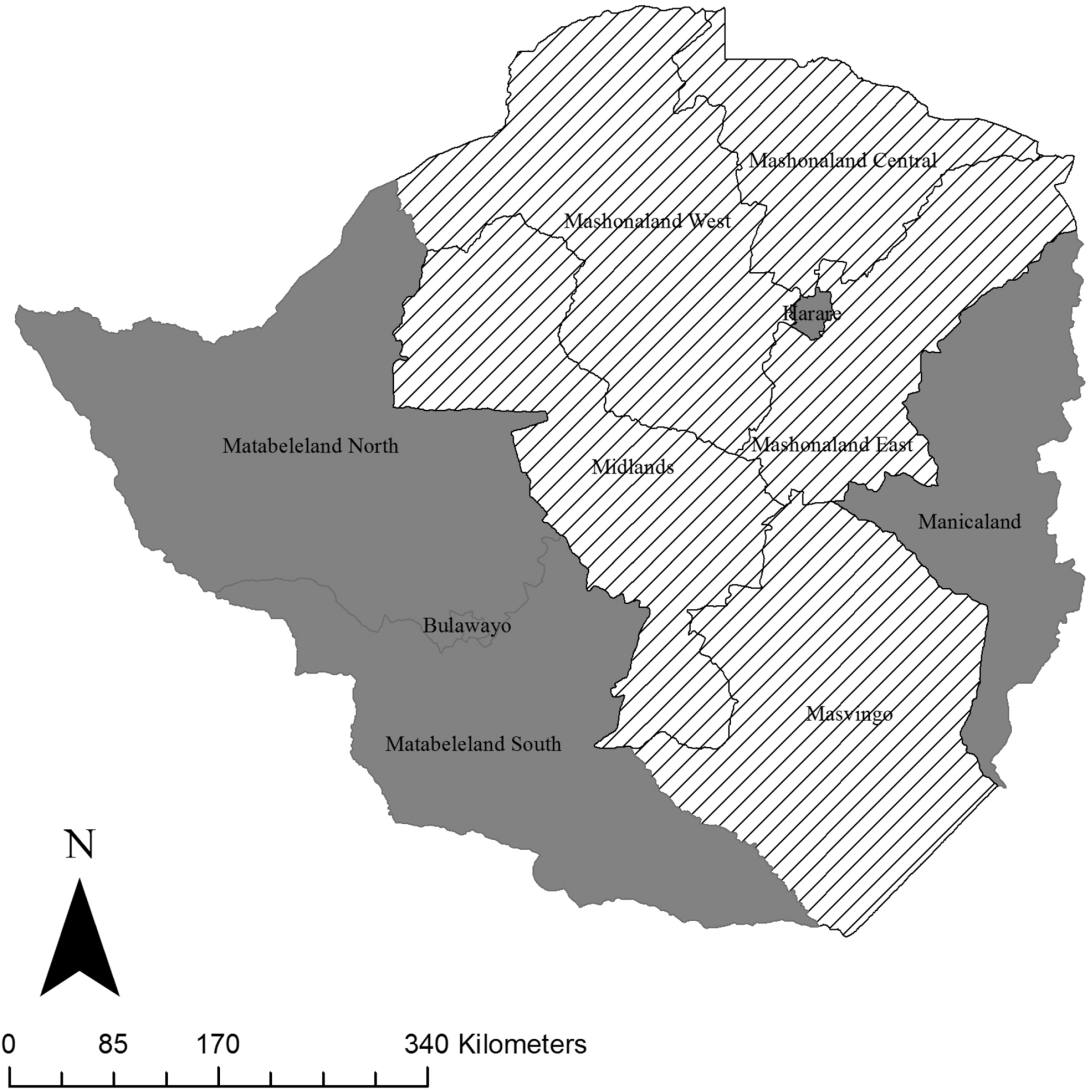
Map of provincial trends of sputum-smear positive (SSP) TB notification rates compared to the national SSP TB notification rate during 2008–2011. *Grey regions* represent provinces for which the average notification rate of SSP TB is above the national average; striped regions represent provinces for which the average SSP TB notification rate of is below the national average

Two of the provinces that had average SSP TB notification rates above the national average, Bulawayo and Harare/Chitungwiza city, are largely urban centers while the remaining three are largely rural [9]. Additionally, with the exception of Harare/Chitungwiza City and Manicaland, these provinces are all located in the western region of the country (Fig. 2).

The provinces that had SSP TB notification rates lower than the national average are all largely rural provinces in the eastern region of the country [9]. Again here, the trend in some of these provinces showed a divergence from the national trend. Mashonaland West had a SSP TB notification rate below the national average in 2009 but slightly above the national average by 2011. In Midlands, the SSP TB notification rate increasingly diverged from the national average; from 2008 to 2010 the notification rate in Midlands tracked with the national average but seemed to be falling at a faster rate than the national average by 2011.

## Discussion

We used aggregated national TB data to assess trends in SSP TB notification rates nationally and by province, age, and sex. Our analysis revealed several findings with important implications for improving TB control in Zimbabwe in the future. First, the SSP TB notification rates were higher in the two main urban provinces, the western provinces and Manicaland. Second, the 25–44 year age group accounted for the largest proportion of notified SSP TB, however, the 55–64 year and 65+ age groups had notification rates in 2011 higher than the 2008 value. Finally, the SSP TB notification rate in males was consistently higher than in females, by an average of 23 %.

The notification rate of SSP TB increased during 2008–2010 and then began sharply declining leading to a lower notification rate in 2011 than what was observed in 2008. During this period there were significant increases in funding to the NTP resulting in improved health system strengthening, including strengthening of laboratory services supported by the Global Fund [10]. Thus, the increase in the national SSP TB notification rate from 2008 to 2010 may not be reflective of an increase in transmission but rather an increase in disease detection resultant from improvements in case finding [10, 11].

Additionally, the harsh economic conditions of the last decade resulted in the deterioration of the public health system in Zimbabwe leading to diminished surveillance systems. As a result, TB data for the period before 2008 was low quality and not used in this analysis. Better quality data became available from 2008 onwards. Thus, similarly the apparent increase in SSP TB case notification rates from 2008 to a peak in 2010 might well be a reflection of the improvement of TB surveillance systems rather than a true increase in the rate of SSP TB. Alternatively, the fluctuation in SSP TB case notification could have been a true change in TB transmission dynamics as a consequence of both internal and external migration. From 2007 to 2008, there was government sponsored internal migration initiating the movement of illegal settlers to less congested rural areas around Harare. Also, concurrently there was external migration of Zimbabweans to neighboring countries in search of employment opportunities. These movements may have increased TB transmission in receiving areas and reduced transmission in areas from which people were leaving.

According to the 2002 Census, 25 % of the population was within the 5–14 age group, 25 % within the 15–24 age group, and 20 % was in the 25–44 age group [9]. However, despite the 25–44 age group accounting for 20 % of the population, this age group accounted for 60 % of the SSP TB cases. The 25–44 year age group has been deemed a higher risk age group in many countries for infection with TB and/or HIV [12]. While the decline in notified SSP TB rates in this age group suggests that control efforts have been effective, this age group should still remain an area of sustained effort. The burden of disease in this age group has serious implications for the population as this age group represents the most active portion of the population in terms of the economic workforce as well as social infrastructure. In addition, this age group represents likely drivers of transmission within the population not only because this age group is more active in the workforce but also because this age group has reported higher levels of sexually transmitted infections and HIV [4]. A sustained TB epidemic in this population could have long-term implications for the health and growth of the population both socially and economically.

The observed low SSP TB notification rates among the 0–4 and 5–14 year age groups could have been due to the difficulty in accurately assessing pediatric TB. These data should therefore be viewed with caution [13].

While the older age groups of 55–64 years and 65+ years do not account for a large proportion of SSP TB cases, these are the only subpopulations in which there were rising SSP TB notification rates. In developed countries an increased incidence rate of SSP TB among the elderly is driven by reactivation of latent TB infection [14]; however, it is unclear whether this same explanation is true in developing countries. Molecular epidemiologic studies could aid in determining whether the notification rate in these age groups is attributable to recent transmission or reactivation of formerly contracted infection. However, such studies are extremely difficult to carry out in a high-burden setting such as Zimbabwe because of the high number of cases that would need to be genotyped for such a study.

The most intriguing finding of the study is the male to female case ratio. In many high burden countries, a disproportionate burden of disease in males with nearly a 2:1 ratio of male to female cases among adults has been observed [3]. For the Africa region as a whole, the WHO reported a male to female ratio of 1.4:1 for SSP TB for both 2010 and 2011 [5, 15]. In our study, the ratio of male to female cases was nearly 1:1, lower than both its neighboring countries and the region.

The provinces that exhibited SSP TB notification rates consistently above the national average are likely epicenters of TB transmission, responsible for the high national TB burden. Harare City is the capital city and has the highest proportion of the national population (16.2 %) and consistently had the highest number of SSP TB cases [2]. Bulawayo, however, has a relatively small population but still had a high burden of SSP TB. There are several possible explanations for why we observe a concentration of SSP TB in these provinces. Both Bulawayo and Harare have higher proportions of the high-risk population, those aged 25–44 years, compared to other provinces. The population density of these urban centers may provide a more hospitable environment for TB transmission to occur. In addition, individuals living in these urban centers often have better access to health facilities including TB diagnostic services. The higher SSP TB notification rates in these urban centers may then partially reflect a higher degree of disease detection.

The aggregation of SSP TB in the western region of the country may be resultant of several factors. The western region of the country historically has a higher HIV notification rate [16] producing higher TB incidence rates. Matabeleland South, for example, has the highest HIV prevalence of 21 %, followed by Bulawayo with an HIV prevalence of 19 % [4]. The high SSP TB notification rate could have been a consequence of increased importation of the disease. The increased migration between the southern provinces and neighboring countries may have resulted in the importation of TB to the Zimbabwean population resulting in higher rates of SSP TB cases compared to national average [17]. Finally, it is possible the higher SSP TB rates may actually reflect a stronger TB program with enhanced case finding and reporting in this region.

The provincial analysis showed that TB control efforts need to focus on urban, high-density areas and rural provinces that are focal points for migration between Zimbabwe and neighboring countries. Provinces exhibiting persistently high SSP TB case notification will require further studies to determine whether the high SSP TB notification is resultant from high TB transmission, reactivation of latent TB infection, better TB detection, or a combination of all of these possibilities. The provinces for which the average SSP TB notification rate was lower than national SSP TB notification rate should also be examined in order to better understand the successes of TB control in these provinces or identify a potential under detection of SSP TB in these regions.

This study had several limitations. First, the nature of the data used for this analysis is inherently limited as it is aggregated and thus inferences cannot be made at the individual level. Further, there were not quality data available regarding HIV status in the TB population limiting our ability to explore HIV as a variable. Additionally, there was a high proportion of sputum-smear not done among the retreatment cases. However, given the retreatment cases only comprised 8 % of the total pulmonary TB cases, we believe that the effect on the study findings was minor.

## Conclusions

Despite the limitations discussed above, this study provides the first detailed description of the TB epidemic in Zimbabwe and has generated data that can provide guidance for future TB control efforts. Findings from this study suggest that TB control has successfully decreased the notification rate of SSP TB in Zimbabwe during 2008–2011. However, the disproportionate distribution of SSP TB among specific regions and subpopulations highlights the need for more targeted interventions in Zimbabwe to accelerate the decline of TB in the country.

## Abbreviations

TB: tuberculosis
AFB: acid-fast bacilli
SSP: sputum-smear positive
HBC: high-burden country
HIV: human immunodeficiency virus
WHO: World Health Organization
NTP: National TB Programme
EPTB: extra-pulmonary TB
SSN: sputum-smear negative
